# Aortic Function’s Adaptation in Response to Exercise-Induced Stress Assessing by 1.5T MRI: A Pilot Study in Healthy Volunteers

**DOI:** 10.1371/journal.pone.0157704

**Published:** 2016-06-16

**Authors:** Laurence Bal-Theoleyre, Alain Lalande, Frank Kober, Roch Giorgi, Frederic Collart, Philippe Piquet, Gilbert Habib, Jean-François Avierinos, Monique Bernard, Maxime Guye, Alexis Jacquier

**Affiliations:** 1 CRMBM-CEMEREM, UMR 7339 CNRS, Aix-Marseille University, Marseille, France; 2 Department of Radiology and Cardiovascular Imaging, La Timone Hospital, Marseille, France; 3 Department of Vascular Surgery and Vascular Medecine, La Timone Hospital, Marseille, France; 4 LE2I, UMR 6306 CNRS, University of Burgundy, Dijon, France; 5 MRI Department, University Hospital of Dijon, Dijon, France; 6 SESSTIM, UMR_S 912 INSERM, Aix-Marseille University, Marseille, France; 7 Department of Biostatistic and ICT, La Timone Hospital, Marseille, France; 8 Department of Cardiac Surgery, La Timone Hospital, Marseille, France; 9 Aortic Center, La Timone Hospital, Marseille, France; 10 Cardiology Department, La Timone Hospital, Marseille, France; Angiology and Arteriosclerosis Diseases, ITALY

## Abstract

**Aim:**

Evaluation of the aortic “elastic reserve” might be a relevant marker to assess the risk of aortic event. Our aim was to compare regional aortic elasticity at rest and during supine bicycle exercise at 1.5 T MRI in healthy individuals.

**Methods:**

Fifteen volunteers (8 men), with a mean age of 29 (23–41) years, completed the entire protocol. Images were acquired immediately following maximal exercise. Retrospective cine sequences were acquired to assess compliance, distensibility, maximum rates of systolic distension and diastolic recoil at four different locations: ascending aorta, proximal descending aorta, distal descending aorta and aorta above the coeliac trunk level. Segmental aortic pulse wave velocity (PWV) was assessed by through plane velocity-encoded MRI.

**Results:**

Exercise induced a significant decrease of aortic compliance and distensibility, and a significant increase of the absolute values of maximum rates of systolic distension and diastolic recoil at all sites (p<10–3). At rest and during stress, ascending aortic compliance was statistically higher compared to the whole descending aorta (p≤0.0007). We found a strong correlation between the rate pressure product and aortic distensibility at all sites (r = - 0.6 to -0.75 according to the site, p<10–4). PWV measured at the proximal and distal descending aorta increased significantly during stress (p = 0.02 and p = 0.008, respectively).

**Conclusion:**

Assessment of regional aortic function during exercise is feasible using MRI. During stress, aortic elasticity decreases significantly in correlation with an increase of the PWV. Further studies are required to create thresholds for ascending aorta dysfunction among patients with aneurysms, and to monitor the impact of medication on aortic remodeling.

## Introduction

Evaluation of the risk of aortic rupture is mainly based on the vessel diameter but this parameter cannot fit all patients [1]. In vivo evaluation of biomechanical properties of the aortic tissue might be of interest to identify patients with a higher risk of aortic events. Arterial stiffness is a marker of vascular health and a predictor of cardiovascular events independent of traditional risk factors. It is caused by structural changes in the vascular wall, including fibrosis, smooth muscle cell apoptosis, and elastic fiber disruption [2,3]. Applanation tonometry evaluation of pulse wave velocity (PWV) is widely accepted as the “gold standard” method for noninvasively assessing arterial stiffness [4–6]. Aortic distensibility appears to be a relevant parameter in premature aortic aging related to genetic familial aortic diseases complicated with thoracic aneurysms and/or dissections in young adults. Other parameters of the aortic wall properties could be the maximum rates of systolic distension (MRSD), and the maximum rates of diastolic recoil (MRDR), both reflecting the elastic modulus of the aortic wall [7]. Cardiovascular magnetic resonance imaging (MRI) has been described as an effective tool to assess aortic strain and distensibility at rest, strongly correlated with PWV, in both healthy and aging populations [8–11] and in patients with Marfan syndrome [12]. Lalande et al. demonstrated that the compliance impairment of the ascending aorta could serve as an early marker of the disease with a discriminative value in asymptomatic patients with MYH11 gene mutation, independent of the presence of aortic dilatation [13,14]. Abnormal functional aortic results could modify patient management as surgical indication is based on aortic diameter (45-50mm for the ascending aorta) and family history [15,16].

Variations of aortic compliance (AC), distensibility (AD), PWV, MRSD and MRDR during exercise are not well-established [17], may be relevant for risk assessment in cardiovascular diseases and have never been studied by MRI along the aorta. Researchers have validated the concept of performing physical exercise during MRI [18], but to date no rest/stress comparisons of the aforementioned parameters using MRI flow measurements were performed. The purpose of this study was to assess regional aortic compliance, distensibility and stiffness during supine bicycle exercise at 1.5 T *(versus at rest)* in asymptomatic individuals with applanation tonometry as standard of reference.

## Materials and Methods

### Study population

The study was approved by the local ethics committee (CPP Sud Méditerranée I, Marseille, ID RCB 2012-A01093-40), and written informed consent was obtained from all subjects. Fifteen volunteers were recruited. Inclusion criteria were: age in between 25 and 40 years, volunteers with no history of cardiovascular disease, neither hypertension, diabetes nor hypercholesterolemia. Height, weight, current smoking status [19] and physical activity (hours/week) were recorded. The exclusion criteria were: contra-indication for MRI, cardioprotective medication and patients with a bicuspid aortic valve diagnosed using MRI. The enrolled subjects performed the protocol after 12 hours of abstinence from smoking and 6 hours from drinking coffee.

### Magnetic resonance imaging

**Fig 1:**
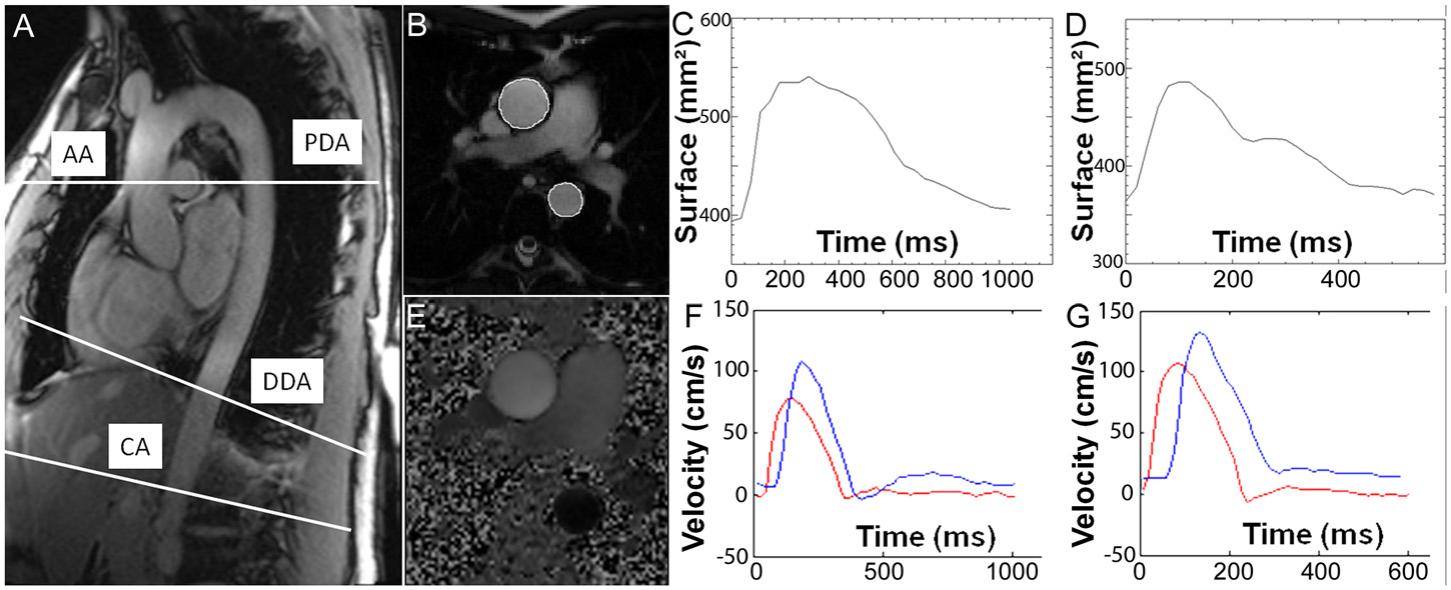
Functionnal MRI methodology. SSFP images in oblique sagittal plane (A) to describe aortic segmentation and lengths at three levels (pulmonary arterial trunk, descending aorta 3 cm above the diaphragm, and aorta above the coeliac trunk), and to focus on four locations (AA, ascending aorta, PDA, proximal descending aorta, DDA, distal descending aorta, CA, aorta above the coeliac trunk). Retrospective cine sequence acquired at the AA and DDA (B) to assess compliance (AC) and distensibility (AD), with respective area profiles during a cardiac cycle, at rest ((C), at the AA level, ΔS = 147 mm^2^, AC = 2.62 mm^2^/mmHg, AD = 6.6.10^−3^ mmHg^-1^) and at peak stress ((D), at the AA level, ΔS = 122 mm^2^, AC = 1.76 mm^2^/mmHg, AD = 4.8.10^−3^ mmHg^-1^). Aortic arch PWV assessed by through plane velocity-encoded MRI (E) at rest ((F), Δt = 61 ms, PWV = 3.89 m/s) and at peak stress ((G), Δt = 57ms, PWV = 4.18 m/s).

All subjects underwent MR in a 1.5T magnet (Siemens, Avanto, Erlangen, Germany) using a 12 channel anterior surface coil array and a 5-element posterior coil array. Three types of sequences were used. All images at rest and during stress were assessed during breath-hold (Fig 1).

1) A stack of segmented steady-state free precession (SSFP) bright blood images were acquired in axial and oblique sagittal planes to assess the aortic segmentation with the following parameters: TR = 161.8 ms, TE = 1.23 ms, α = 80°, FOV = 340×340 mm^2^ (for axial orientation), pixel size = 1.33×1.33 mm^2^, slice thickness = 6 mm.

2) SSFP cine images were acquired at three different levels: pulmonary trunk, 3 cm above the diaphragm at the distal descending aorta (DDA), and above the coeliac trunk (CA). The imaging plane was carefully oriented to be perpendicular to the aorta at each level. The imaging plane at the level of the pulmonary trunk was positioned so that the ascending aorta (AA) and the proximal descending aorta (PDA) could be measured on the same image. Difference in angulation between ascending and descending aorta never exceeded 15°. The following parameters were used (with variation depending on the subject): TR = 21.7 ms to 24.7 ms, TE = 1.36 ms to 1.55 ms, α = 65°, recFOV = 210×263 mm^2^ to 280×340 mm^2^, slice thickness = 7 mm, pixel size = 1.26×1.26 mm^2^ to 1.68×1.68 mm^2^, iPAT (acceleration factor 2). According to the patient heart rate, 30 to 40 phases were reconstructed. Before and after each image acquisition, the arterial diastolic and systolic blood pressures at rest were measured using a brachial automatic sphygmomanometer (Maglife, Schiller Medical).

3) Velocity encoding gradient pulse sequences were acquired at the same plane as the cine images with a velocity encoding gradient in through plane direction. The following parameters were used: maximum encoding velocity 200 cm/s, TR = 28.35 ms, TE = 2, α = 30°, slice thickness = 6 mm, recFOV = 192×280 mm to 250×365 mm^2^ (depending on the subject, corresponding to a pixel size of 1.46×1.46 mm^2^ to 1.88×1.88 mm^2^), 60 reconstructed phases. For the estimation of the time difference between arrival of the pulse waves at two different levels of the aorta, we considered the slope at the beginning of the systolic phase [20]. The lengths of the aortic segments were calculated from the anatomic SSFP slices by indicating the center of the aorta on each slice between two levels of interest [21]. Then the PWV was calculated for the aortic segments between AA and PDA (^AA/PDA^ PWV), between AA and DDA (^AA/DDA^ PWV) and between AA and CA (^AA/CA^ PWV).

### Supine bicycle exercise in the MRI room

**Fig 2:**
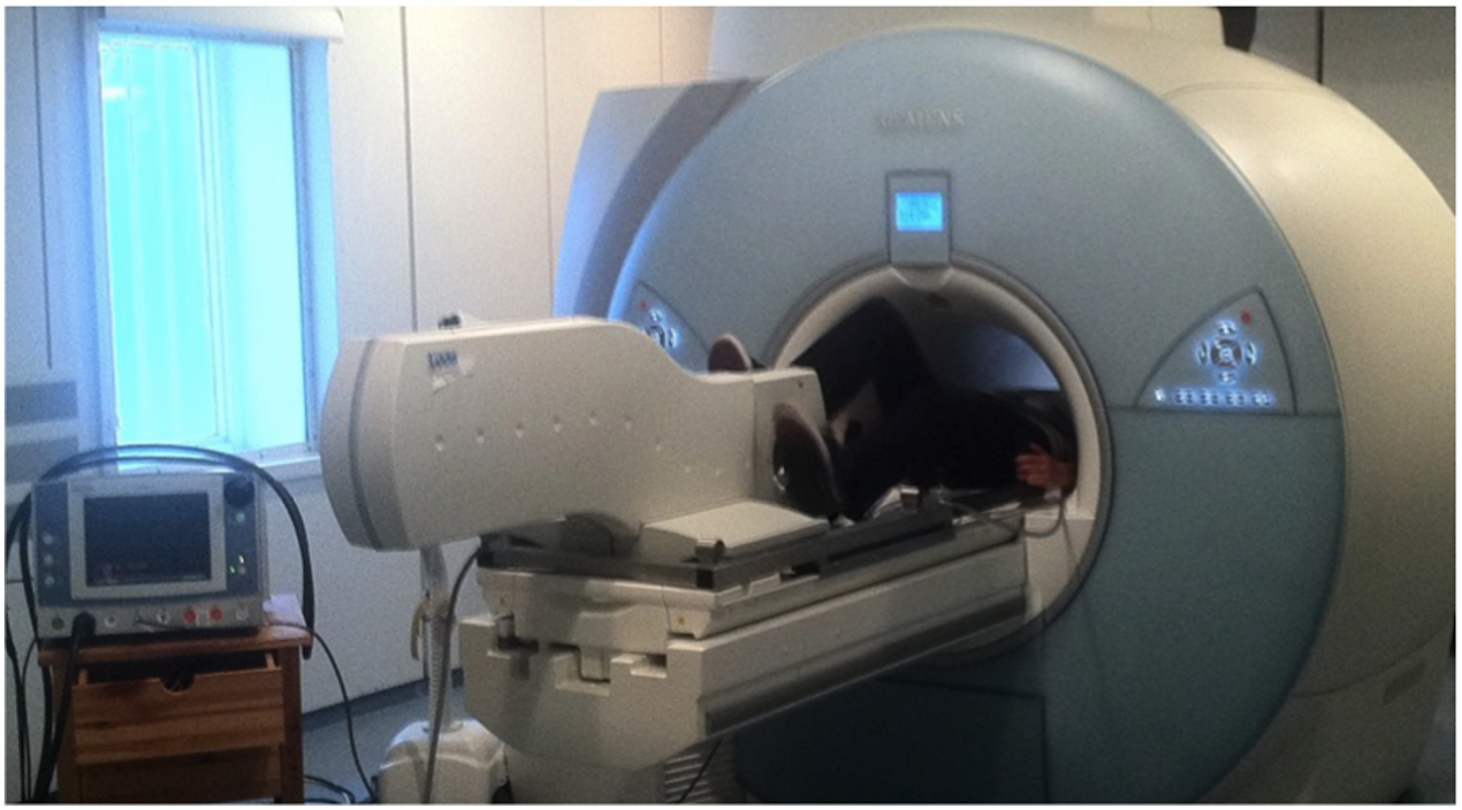
Amagnetic ergometer (LODE, Netherlands) allowing a supine treadmill test during MRI (1.5 T).

**Fig 3:**
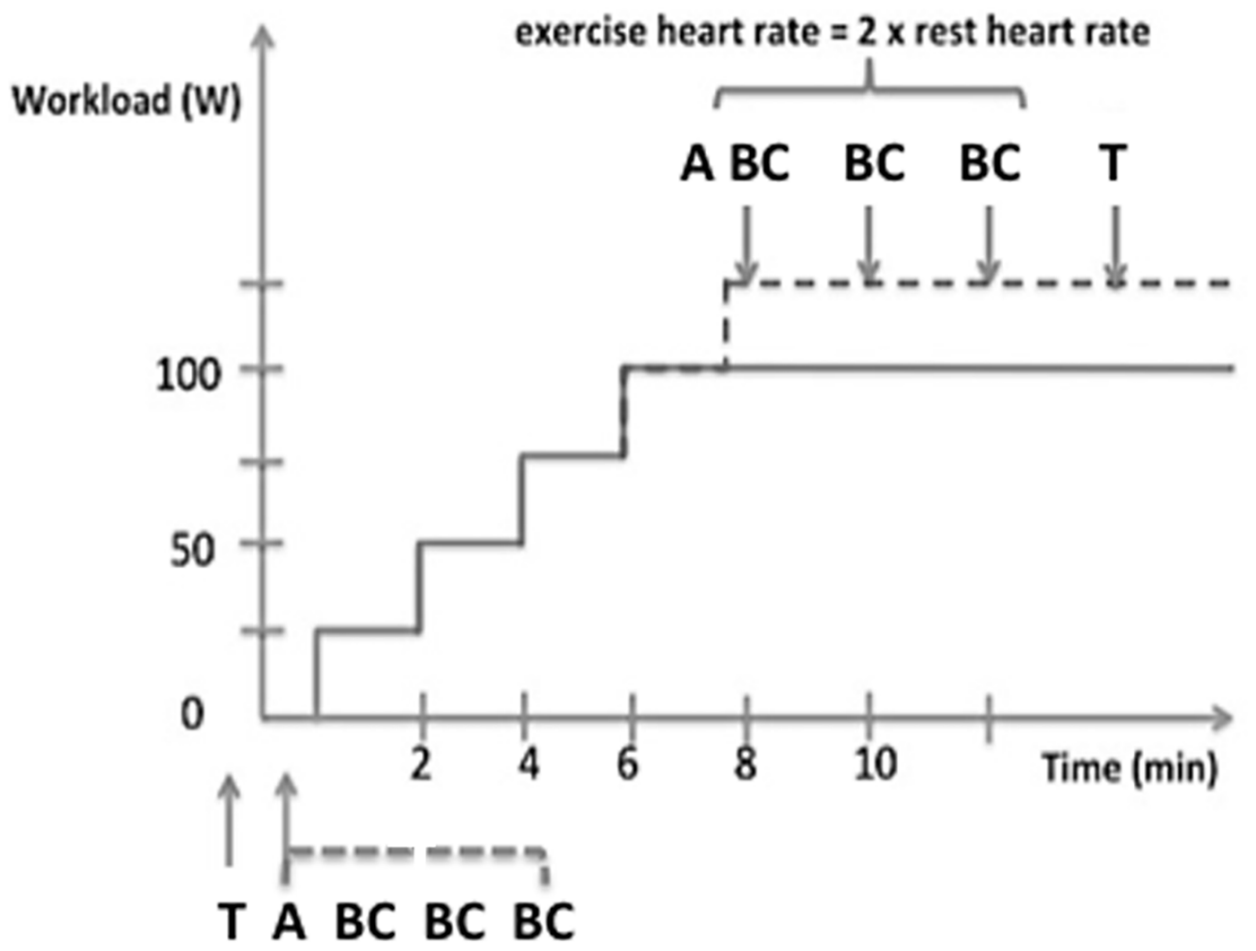
MR sequences during supine bicycle exercise-induced stress protocol. A: True-FISP sequence, B: Cine FISP sequence, C: Gradient pulse sequence, T: Tonometry. Both Cine FISP and gradient pulse sequences were acquired successively at three levels, at rest and at peak stress. Tonometry was performed at rest and at the end of a second bicycle exercise under the same protocol, monitored at least half an hour after the first one.

For physical exercise, an amagnetic ergometer (Lode, the Netherlands) was fixed to the table of the MRI system (Fig 2). The position of the ergometer’s foot pedals was adjusted to the height of the subject. The heart rate was measured by electrocardiography (ECG). The training mode of the ergometer allowed us to adjust the workload in Watt and was independent from pedal rotation per minute, as previously described [18]. The volunteers were instructed to cycle for two minutes at 25 W, workload was increased by 25 W every two minutes for a minimum duration of ten minutes, such as to obtain twice the resting heart rate. This way, an appropriate stress level to make clinical relevant observations could be achieved. The volunteer was then instructed to stop cycling, and was positioned at the center of the magnet. As the heart rate and blood pressure diminished quickly after stopping cycling, we decided to acquire only two datasets of images immediately after cycling was stopped, corresponding to cine- and velocity-encoding images for one aortic level. The approximate delays between the cycling interruption and the acquisitions were less than 1 minute for both acquisitions. The arterial blood pressures during exercise were acquired before and after these two acquisitions (and the average was considered). The volunteer was then moved out of the magnet just enough so that they could restart cycling. They were then instructed to cycle for at least two minutes at the last exercise level. The image acquisition was repeated until the protocol was complete (Fig 3).

### Data post processing

Data were analyzed off-line using the QIR scientific image analysis software (Université de Bourgogne, Dijon, France) [13]. The contours of the aorta were automatically traced for all phases of the cardiac cycle (Fig 1). This processing was performed on SSFP cine images because these images provided high contrast between aortic wall and lumen with manageable artifacts due to flow. User interaction was limited to the indication of only one point near the center of the aorta on the first image. This automatic method of vessel wall contour segmentation has already been described in detail previously [21]. In summary, for each image, there is firstly a filtering by a Haralick filter following smoothing with a Gaussian filter and a contour enhancement step, followed by an extraction of the aortic wall contour based on a graph searching technique in a polar representation of the data. The AC is the absolute change in aortic area (Δarea) for a given pressure step (ΔP) (AC = Δarea/ΔP, in mm^2^/mmHg). Considering the area instead of the diameter rendered the calculation of the AC more robust to variations due to spatial resolution. The maximum and minimum areas were obtained from cross sectional area versus time curves. AD was defined as the compliance divided by the minimum cross-sectional area, and expressed in mmHg^-1^. Brachial pulse pressure (in mmHg) was defined as [systolic blood pressure—diastolic blood pressure] and two different values were considered for rest and exercise. MRSD and MRDR were defined as the maximum systolic upslope and the maximum diastolic downslope [7] and expressed in mm^2^/ms (absolute values).

### Tonometry data acquisition and analysis

Rest tonometry was performed in supine position before MRI following a 10-min period of rest. We used a commercially available device (SphygmoCor CVMS, AtCor Medical, Sydney, Australia) customized to record all physiological signals including ECG and tonometric signals from right common carotid and right femoral arteries, with highly reproducible results in both healthy and diseased populations. Tonometry data were acquired using the recommended methodology [6]. Carotid-femoral pulse wave velocity (cfPWV) was calculated from the distance between arterial recording sites divided by the pulse transit time: D/Δt. Tonometry under stress condition was performed after completing a second exercise protocol with a standard ergometer.

### Statistical analysis

Continuous data with a normal distribution are expressed as mean±SD and non-normal distributed data as median (range). Categorical data are expressed as frequency or percentage.

A Friedman test was used to compare AC’s and AD’s heterogeneity along the aorta under each condition (rest or exercise). A Wilcoxon test with Bonferroni correction was used to compare the different values for regional AC and AD, under each condition (rest or exercise). A Wilcoxon test for paired regional series (rest versus exercise) was used to analyze the effect of stress on regional AC and AD. Correlation between regional distensibility and cardiac product was performed with a Spearman test. A p-value smaller 0.05 was considered to represent statistical significance. Comparison between tonometry and PWV measured with MRI was also performed with the calculation of the correlation coefficient and with a Bland Altman test (applanation tonometry being the reference method).

## Results

### Study population and exercise data

Baseline characteristics of the fifteen healthy volunteers are shown in Table 1. There was no difference between volunteers except for a light smoking status for five of them (1.33 PA (0–5)). Aortic dilatation or aortic valvulopathy were not detected in morphological analysis and the left ventricular function was normal in each participant. All volunteers completed the entire protocol at the predicted cardiac performance (Table 2). Brachial pulse pressure (PP) at rest was in accordance with data from Redheuil et al. (50±7 mmHg vs. 48±7 mmHg) [10].

**Table 1:** Baseline characteristics of the volunteers.

**Volunteers (n)**	**15**
**Mean age (years (min-max))**	29 (23–41)
**Men/women (n)**	8/7
**Tobacco consumption (n (%))**	5 (33)
**Mean BMI (kg/m^2^ (min-max))**	20 (18–25)
**SAP (mmHg)**	122.2±9.31
**DAP (mmHg)**	71.1±4.8
**Sport > 1h/w; n(%)**	8 (53)
**Familial aortic disease (n)**	0

(BMI: Body mass Index, SAP: Systolic arterial pressure, DAP: Diastolic arterial pressure)

**Table 2:** Effects of exercise on hemodynamics during MRI and tonometry protocols.

	Rest MRI	Exercise MRI	Rest tonometry	Exercise tonometry
**MAP**	91±9	104±7*	92±10	106±10^$^
**PP**	50±7	74±11*	56±9	82±16^$^
**HR**	69±9	127±17*	67±7	102±12^$^

(PP: pulse pressure (mmHg), MAP, mean arterial pressure (mmHg), HR: heart rate (bpm))

*:p <0.05 for rest versus exercise MRI;

^$^:p<0.05 for rest versus exercise for tonometry.

### Regional functional parameters by MRI

#### At rest

**Fig 4:**
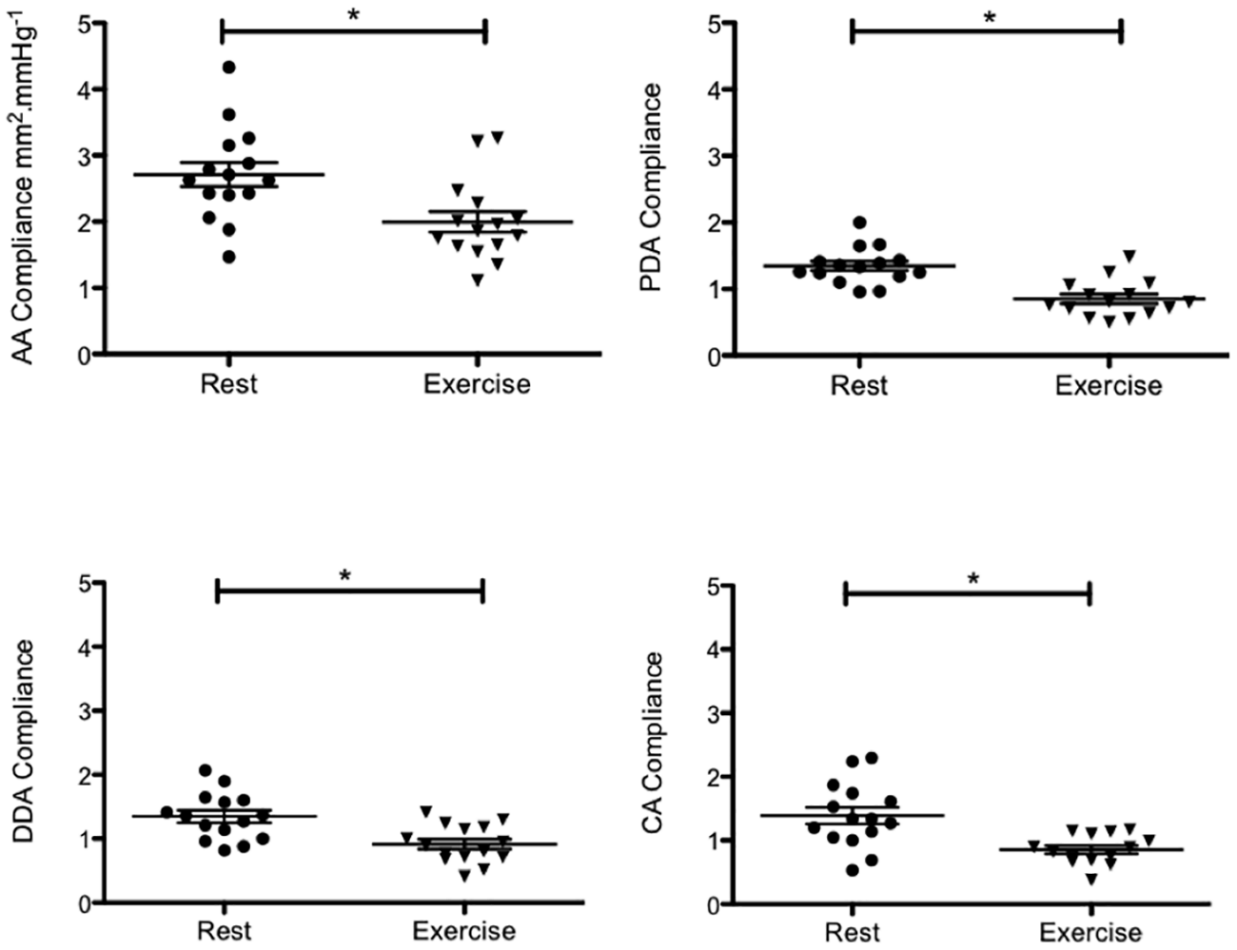
Regional aortic compliance at rest and under exercise conditions (n = 15; *p≤0.0007).

AC (Fig 4) and AD were statistically higher in AA compared to the whole descending aorta (p≤0.0007), which demonstrates the heterogeneity of elastic properties along the aorta. Segmental aortic PWV (^AA/PDA^ PWV, ^AA/DDA^ PWV and ^AA/CA^ PWV) values did not differ from each other (Table 3).

**Table 3:** Regional variation in pulse wave velocity at rest and under exercise conditions (mean±SD, m/s). Comparison between MRI and tonometry (correlation coefficient and Bland Altman test (mean± SD, m/s)).

	^AA/PDA^ PWV_MRI_	^AA/DDA^ PWV_MRI_	^AA/CA^ PWV_MRI_	cf PWV
**Rest PWV**	4.7±1.4	3.9±0.5	3.9±0.6	5.4±0.8
**r**	0.41^$^	0.22^$^	0.06^$^	
**Bland Altman test**	-0.84±1.25	-1.46±0.8	-1.41±0.93	
**Exercise PWV**	5.6±1.3*	4.8±1.2*	4.2±1.2	5.5±0.7
**r**	0.43	0.08^$^	0.13^$^	
**Bland Altman test**	0.12±1.17	-0.65±1.33	-1.21±1.29	

(CA: coeliac trunk, DDA: Distal descending aorta, PDA: Proximal descending aorta, AA: Ascending aorta, PWV: Pulse wave velocity, cf PWV: carotid-femoral PWV by tonometry, r: correlation coefficient between MRI and tonometry)

*:p<0.05 for rest *versus* exercise PWV_MRI_;

^$^:p<0.05 for MRI versus tonometry.

#### During physical exercise

**Fig 5:**
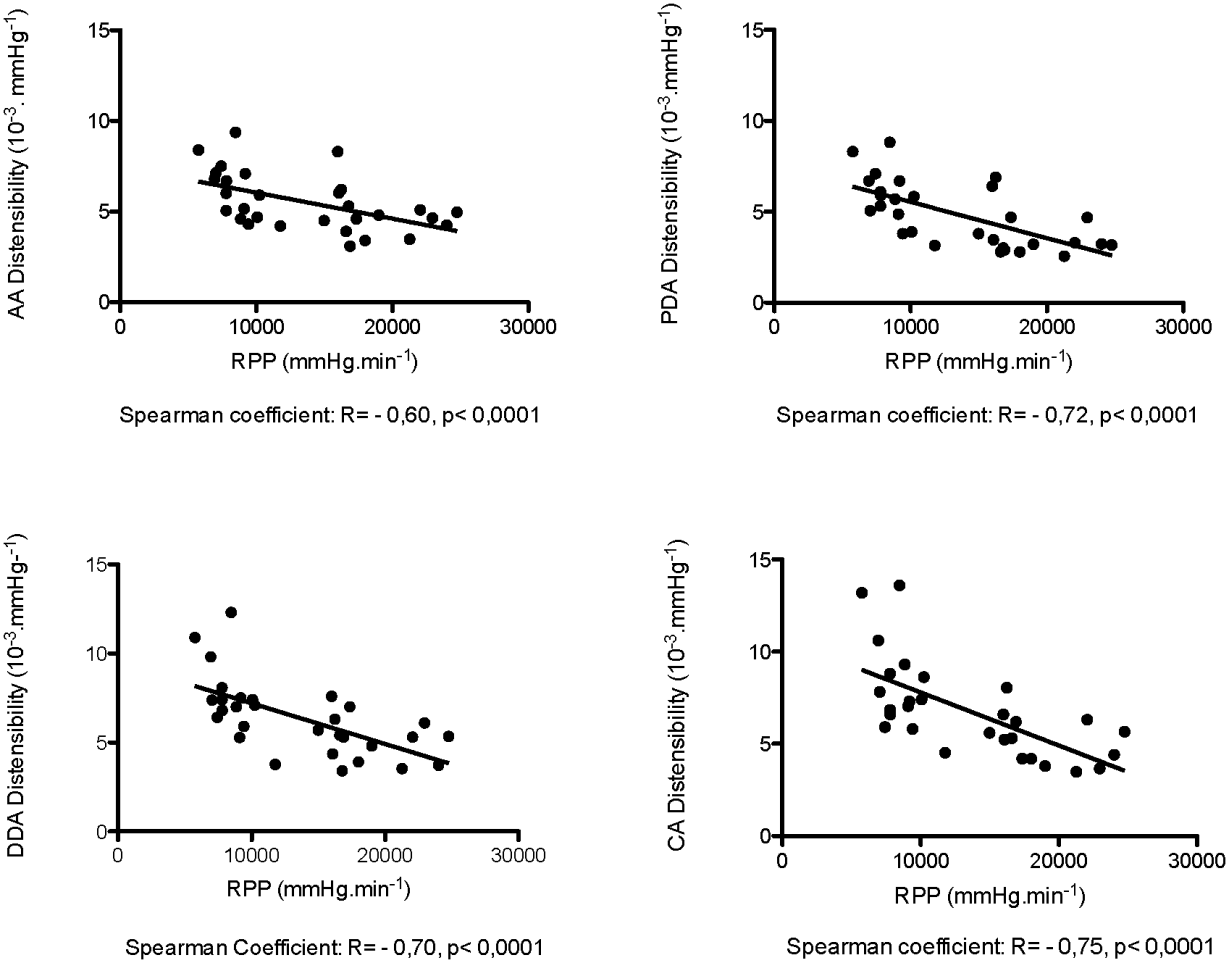
Correlation between regional aortic distensibility and cardiac work. The regional heterogeneity of aortic distensibility (AD) at rest is still relevant at peak stress (medians at rest and at peak stress in mmHg^-1^, ^AA^AD = 6 (4.88–7.12) to 4.64 (4.08–5.2), ^PDA^AD = 5.83 (4.96–6.7) to 3.23 (2.95–4.24), ^DDA^AD = 7.38 (6.6–7.79) to 5.3 (4.13–5.9), ^CA^AD = 7.4 (6.72–9.05) to 5.27 (4.2–6.06)).

**Fig 6:**
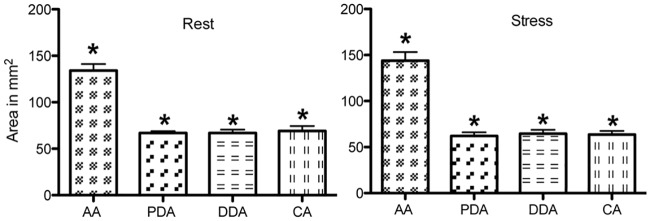
Variation in regional delta area at rest and under exercise conditions. AA delta area was significantly higher than in PDA, DDA, CA under the both conditions (*p<0.001). No difference was observed along the descending aorta.

Stress induced a significant decrease of AC and AD at all sites. Medians for aortic compliance (in mm^2^/mmHg) are as follows (rest versus exercise): ^AA^AC: 2.63 *vs*. 1.86, ^PDA^AC: 1.33 *vs*. 0.8, ^DDA^AC: 1.36 *vs*. 0.9, ^CA^AC: 1.34 *vs*. 0.86, p<10^−3^ (Fig 4). We also found a strong correlation between the rate pressure product and AD at all sites (Fig 5). Furthermore, during exercise, we measured a reduction of the maximal and minimal aortic areas along the aortic arch (Table 4), with a higher and preserved significant delta area in AA versus in PDA, in DDA and in CA (Fig 6). Only ascending aortic strain increased during stress (^AA^strain median *vs*. ^PDA^strain median: 0.37 vs 0.27, p = 0.009). Moreover, there was an increase in the absolute values of MRSD and MRDR for all sites in all volunteers (Table 5).

**Table 4:** Regional variation in maximal and minimal aortic areas (mean, mm^2^) at rest and under exercise conditions.

	Max aortic area			Min aortic area		
	rest	exercise	p	rest	exercise	p
**AA**	581.3	563.4	0.07	447.2	421.5	0.002
**PDA**	301.2	289.8	0.009	240.3	229.4	0.01
**DDA**	250	240.8	0.11	183.1	177.3	0.13
**CA**	241.3	232.8	0.14	172.3	169.2	0.36

(AA: Ascending aorta, CA: coeliac trunk, DDA: Distal descending aorta, PDA: Proximal descending aorta)

**Table 5:** Regional variation of MRSD and MRDR (mean ± SD, mm^2^/ms) at rest and under exercise conditions.

	MRSD		MRDR	
	rest	exercise	rest	exercise
**AA**	2.03±0.77	4.51±4.00	-0.71±0.19	-1.39±0.39
**PDA**	0.88±0.22	1.49±0.40	-0.36±0.07	-0.61±0.11
**DDA**	0.81±0.21	1.42±0.36	-0.38±0.10	-0.62±0.16
**CA**	0.94±0.35	1.35±0.37	-0.44 ±0.18	-0.75±0.16

(AA: Ascending aorta, CA: coeliac trunk, DDA: Distal descending aorta, MRDR: Maximal rate of diastolic recoil, MRSD: maximal rate of systolic distension, PDA: Proximal descending aorta)

p<10^−4^ for all between rest and exercise.

PWV measured at PDA and at DDA increased significantly during stress (Table 3), in correlation with the decrease of aortic elasticity. No segmental difference in PWV (^AA/PDA^ PWV, ^AA/DDA^ PWV and ^AA/CA^ PWV) was observed along the aorta during physical exercise.

### Carotido-femoral PWV by Applanation Tonometry

#### At rest

CfPWV at rest (5.4±0.76 m/s) was in accordance with normal range data previously published describing young populations without cardiovascular risk factors (in a multi-centric study on 1455 healthy subjects, cfPWV was estimated at 6.2±0.75 m/s for subject less than 30 years, and at 6.5±1.35 m/s for subject between 30 and 39 years old) [22], and higher than PWV measured at PDA, at DDA and at CA by MRI (Table 3), as shown by the Bland Altman tests. The correlation coefficient is low between cfPWV and PWV measured at PDA (aortic arch PWV), and weak for the two other sites (Table 3).

#### During physical exercise

No significant difference was measured in cfPWV when comparing stress to rest. According to Bland Altman tests, cfPWV was still higher than aortic PWV at DDA and at CA by MRI, but with a very little bias at the level of the PDA. Again, the correlation coefficient is low between cfPWV and PWV measured at PDA (aortic arch PWV), and weak for the two other sites (Table 3).

## Discussion

Although previous studies were focused on the effect of cardiovascular risk factors on aortic stiffness, we described the effect of exercise on aortic compliance and distensibility in a young healthy volunteer group [23–25]. To achieve this goal, image acquisition was initiated immediately after peak exercise to ensure that peak stress biomechanic changes were captured and completed within the first minute after peak exercise for each aortic level. Both compliance and distensibility decreased significantly during physical exercise, in correlation with an elevation of PWV. It seems to be due not only to the increase of PP during physical exercise, but also to a significant reduction—or an absence of increase- of maximal and minimal aortic areas along the aorta. This latter point has been previously described by Weber et al. at the ascending aorta and the pulmonary artery levels by functional MRI during exercise but has never been confirmed so far [18]. Two hypotheses could explain these unexpected observations 1) a constrictive response of aortic smooth muscle cells (i.e. wall tension) during rapid volume expansion 2) a faster MRSD and a faster MRDR during exercise compared to rest, reflecting the elastic reserve of the aortic wall and its adaptation to uptake kinetic energy of systolic ejection and to release pulsatile flow. In our study among healthy volunteers, the absolute values of these parameters increased, confirming this hypothesis. This second hypothesis has been demonstrated at rest by Aquaro et al. who studied patients with bicuspid aortic valves: MRSD were slower regardless of the diameters of the ascending aorta in comparison with controls, indicating an alteration of elastic wall properties [7].

In addition, using our semi-automatic software to measure regional aortic delta area, we have been able to describe a regional heterogeneity in aortic biomechanics during stress and at rest. Distensibility of AA and PDA were significantly higher compared to distal segments even at peak stress. To our knowledge, these MR-variations of distensibility at four levels along the aorta have been described at rest by Kim et al. [26], but never during physical exercise. Only AA presented a slight but statistical increase of relative delta area during stress, which could demonstrate its specific elastic reserve. As premature aortic dilatation in genetic familial diseases occurs mainly in AA, our opinion is that the impaired distensibility of the AA under stress could be an earlier and discriminant marker of aortic stiffening in young patients without dilatation.

One limitation of our study is the estimation of simultaneous brachial PP using a brachial artery sphygmomanometer whereas estimation of central pulse pressure by tonometry is recommended as the most accurate method. Indeed, the calibration of the system could be biased, and changes could appear between measurements in and out of the MRI room. Nevertheless, our method is the only one available to non-invasively evaluate hemodynamic parameters simultaneously with the cine-MRI sequence (two measurements, before and after each acquisition). In consequence, our analysis appeared to induce a slight underestimation of regional distensibility at rest, compared to the study of Redheuil et al. (^AA^distensibility 6.61±2.5.10^−3^ vs. 7.4±2.3.10^−3^, ^PDA^distensibility 6.12±2.3.10^−3^ vs. 7.2±1.8. 10^−3^, in mmHg^-1^) [10]. One of the advantages of the PWV compared to AC is that it is independent of the blood pressure measurement, which could vary slightly during exercise.

As far as aortic PWV is concerned, our results at rest were in line with previous data, as the significant difference compared to cf PWV values [10]. We observed its expected increase at peak exercise, with no segmental difference between aortic arch PWV (^AA/PDA^ PWV_MRI)_ and distal aortic PWV (^AA/CA^ PWV_MRI_). The young age of our subjects is probably the main explanation, as PWV has been described to increase with age until 50 years [24].

It is also important to consider the method used to quantify transit time as there is no established reference method. Crucial points for the calculation of this value are the temporal resolution of the acquisition sequence and the considered part of the curve. Firstly, due to the temporal resolution, MRI could be limited with up to approximately 20 ms imprecision concerning transit time (*Δt*). Moreover, the considered part of the curve (foot of the wave, half of the maximum value or the whole curve) could produce different values. It is generally accepted to use the upstroke as the reference point (i.e. the foot of the wave) [6,20]. In our study, we compared *Δt* with foot-to-foot measurement to *Δt* calculated as the average time difference using the least squares estimate between all data points on the systolic upslopes of the ascending and descending aortic flow curves after peak flow normalization. We demonstrated no significant variation in *Δt* at rest and at peak exercise. Lastly, there was a longer delay between peak stress and tonometry than between peak stress and MRI (>3 minutes versus <1 minute) due to the tonometry protocol. This could explain the absence of significant variation in cfPWV at rest and during exercise.

Functional MRI optimized by exercise-induced stress may give insights into aortic biomechanic adaptation in patients with cardiovascular disease (systemic hypertension, atherosclerosis), thoracic aortic diseases (aneurysms, dissection), or valvular diseases [27–29]. Presymptomatic subjects with Marfan syndrome or related diseases or bicuspid aortic valves are usually under preventive programs based on medical therapy, limited exercise and regular aortic anatomic imaging. Evaluation of the aortic “elastic reserve” (i.e. Z-score for AA distensibility, depending on the measured AD and the estimated mean AD by age, or parameters such as MRSD or MRDR) or the aortic stiffness (i.e. aortic arch PWV) under exercise could provide earlier identification of pathologic aortic remodeling than diameter measurements, and allow an improved personalized therapy [30–32]. Teixido-Tura et al. recently proposed a Z-score for AA distensibility at rest <-3.5 to distinguish Marfan patients from controls with 82.5% sensitivity and 86.1% specificity after adjustments for age, PP, and aortic dimensions [33]. This diagnostic benefit could be optimized during exercise. A recent phase III clinical trial by Laurent et al. focused on the ability of cfPWV to reflect the dose-dependent impact of olmesartan on arterial de-stiffening among hypertensive patients [34]. Segmental aortic PWV should be a more discriminant parameter among patients with cardiovascular diseases -compared with matched controls- than in our group of healthy and young volunteers. Further studies are required to assess the interest of functional aortic parameters at rest and during stress for risk assessment and preventive follow-up strategies in cardiovascular diseases.

## Conclusion

To conclude, the main findings of our study are:

1. The measure of aortic compliance, distensibility, MRSD and MRDR during a supine bicycle-induced exercise is feasible;
2. During stress, aortic elasticity decreases significantly in correlation with an increase of the PWV;
3. There is a regional heterogeneity in aortic biomechanics at stress and at rest, revealed by a significantly higher distensibility of AA and PDA compared to distal segments.

## Supporting Information

S1 Data (RAR)
